# Real-time monitoring of a circulating vaccine-derived poliovirus outbreak immunization campaign using digital health technologies in South Sudan

**DOI:** 10.11604/pamj.2021.40.200.31525

**Published:** 2021-12-01

**Authors:** Isah Mohammed Bello, Maleghemi Sylvester, Melesachew Ferede, Godwin Ubong Akpan, Ademe Tegegne Ayesheshem, Michael Nzioki Mwanza, Samuel Okiror, Atem Anyuon, Olu Olushayo Oluseun

**Affiliations:** 1 World Health Organization (WHO), Inter-Country Support Team Office for East and Southern Africa, Harare, Zimbabwe,; 2 World Health Organization (WHO) Country Office, Juba, South Sudan,; 3 World Health Organization (WHO), Regional Office for Africa, Cite de Djoue, Brazzaville, Congo,; 4 Ministry of Health, Juba, South Sudan

**Keywords:** Geographic information system, circulating vaccine derived poliovirus, cVDPV, vaccination, open data kits, Power BI

## Abstract

**Introduction:**

the use of digital health technologies and geographical information systems (GIS) in the conduct of immunization campaigns had proven to be a success story, and is gaining acceptance towards improving supervision, accountability, and real-time access to quality information. The demand for real-time information by policymakers and stakeholders in the polio eradication programme is increasing towards ensuring a world free from all polioviruses. This study aims to develop a tool that monitor and evaluate the circulating vaccine-derived poliovirus (cVDPV) campaign processes in real-time using open data kits (ODK) to collect data, analyze and visualize using an interactive dashboard in Power BI, towards improving timeliness and completeness of data reporting and providing real-time quality information to stakeholders.

**Methods:**

electronic checklists were developed using open data kits (ODK) and uploaded onto android-based smartphones for data collection during a round of cVDPV outbreak response immunization. Supervisors were deployed to the field and the checklists were utilized at both stages of the campaign activities. A Power BI data visualization tool was used for reporting, analysis, and monitoring the activities of the campaign.

**Results:**

an interactive dashboard was developed, providing real-time information that supports stakeholders during the campaign processes with improved timeliness and completeness of data reporting. The usage of the tool during the campaign enhanced close supervision, and increased transparency in data availability and accessibility by all partners.

**Conclusion:**

the study had shown that real-time information has significantly improved the smooth conduct of the immunization campaign processes through identifying gaps, and challenges in the field and can be utilized in similar resource settings including complex and humanitarian. It has demonstrated the capability of mobile phones using ODK for data collection and linked to a Power BI dashboard for enhanced supervision and transparency, and we encourage further studies to assess the effects of the tools on the campaign results.

## Introduction

The African Region has been certified wild polio-free by the African regional certification commission (ARCC). In August 2020 after the acceptance of documentation from 47 African countries, representing over 90% of the world´s population becoming wild polio-free, with five out of the six (5/6) region certified [1]. However, the battle is not over, as there are outbreaks of circulating vaccine-derived poliovirus (cVDPV), occurring mainly in places with low immunization coverage, most especially in conflict areas and remote communities experiencing huge migration. In the African Region, a total of 380 and 82 cases were confirmed from acute flaccid paralysis (AFP) and environmental surveillance respectively in seven (7) countries as of December 5^th^, 2020. South Sudan had reported 21 cVDPV in 2020 from AFP cases, with 1 case from environmental surveillance in 9 out of the 10 states of the country same period [2]. This has become a challenge to the global polio eradication programme in its final stage of eradication efforts [3]; the silver lining is that the same techniques used to eradicate the wild poliovirus, such as enhancing polio surveillance networks and achieving high vaccine coverage needed to cease transmission, may be utilized to combat the cVDPV2, as outlined in the recently released guide [4].

Digital health technologies use platforms for computing, software, connectivity, and sensors poised for health care and its related usages, it spans a wide range of uses, which includes applications in general wellness and medical device. The general scope of digital health includes among others mobile health (mHealth), telehealth and telemedicine, health information technology (IT), personalized medicine and wearable devices, etc. [5]. The use of these technologies has changed the way we communicate, and also provided innovative ways for monitoring our health and well-being through greater access to information, new options for enhancing prevention, early detection and diagnosis of life-threatening diseases, the extension of life expectancy through management of chronic conditions, etc. [6].

The need for a high-quality campaign is becoming increasingly important with much focus on the use of technologies and geographical information systems (GIS) to support, guide, and monitor the conduct of the campaign activities, towards enhancing close supervision, accountability, transparency, and increasing real-time access to information. The demand for quality and real-time information using geographic information systems (GIS) and other technologies by policymakers at levels by different stakeholders in the polio eradication programme is increasing and several approaches and tactics had been adopted to meet these demands. Digital technologies had proven to have supported positively the polio eradication programme through enhancing surveillance performance [7-10], support the implementation of real-time supplemental immunisation activities (SIAs) [11-13], use of mobile phones to support polio vaccination [14], tracking vaccination teams during the conduct of polio campaigns in northern Nigeria [15], creating maps using a geographical information system to aid microplanning for enhanced close supervision [16], the use of mobile phones short text messages (SMS) for community mobilization and conduct of surveys to support polio campaigns [17,18], use of real-time data transmission approach in campaign monitoring [19], the use of mobile data collection tools in the conduct of lot quality assurance sampling (LQAs) to support post-campaign monitoring [20] and leveraging the use of mobile technology in support of polio post-campaign evaluation [21] among others.

Although there are several efforts in documenting the use of mobile and GIS technologies in the conduct of polio immunization campaigns across different geographical settings, limited studies exist targeting the entire component of the campaign (pre, intra, and post implementation) and using complex and humanitarian settings like South Sudan, which requires more robust and flexible solutions due to its peculiarities like insecurity, high numbers of displaced persons, lack of communication and internet in many parts of the country, etc, with nearly over 4 million people displaced, which includes 1.6 million internally displaced people (IDPs) and 2.2 million South Sudanese refugees [22]. This study is aimed at developing a tool that will monitor and evaluate the campaign processes in real-time, from pre-implementation activities (training, pre-validation, etc.), intra-implementation (team supervision, in-process (inside and outside) monitoring, and post-implementation (post-campaign evaluation, LQAs, and administrative coverage data)) using open data kits (ODK), linked to an interactive dashboard in Power BI. It will also evaluate the impact of the tool on the timeliness and completeness of data reporting and providing real-time information to stakeholders at all levels to take informed action while increasing transparency and accountability.

## Methods

**Study settings:** South Sudan is a landlocked country located in the Sahel Region of East Africa bordering Ethiopia to the east, Kenya to the southeast, Uganda to the south, the Democratic Republic of the Congo to the southwest, the Central African Republic to the west, and Sudan to the north. The country covers a landmass of approximately 644,329 km^2^. The project population based on the 2008 census was estimated to be 13.3 million (2019) using a 3% growth rate. The people of South Sudan have continued to experience the impacts of years of conflict and violence, with limited or no development investment. It had been estimated that about 7.3 million people are facing problems related to their mental and physical well-being [23,24]. Infrastructural development is limited, the country is among the most underdeveloped in terms of road networks in the world, since most of the national, interstate, and urban roads are bad and not maintained, and 60 percent of the limited road network are inaccessible during the rainy season [25].

In response to the cVDPV type 2 outbreak declared on the 18^th^ of September 2020 by the national ministry of health (MOH), the country conducted the first round of campaigns in six (6) states and 44 counties with mOPV2 targeting all children under the age of five (5). The ministry of health alongside other partners (WHO, UNICEF, Core Group, etc.) supported the campaign. The World Health Organization (WHO) provided technical and financial support on training, microplanning, intra-campaign monitoring and supervision, LQAs, and administrative data collection all over the country, United Nations Children's Fund (UNICEF) supported vaccine management, logistics, communication, and social mobilization. Training was conducted at both national, state, and counties level with the support of WHO, UNICEF, and MOH personnel at both levels on the use of the tools, and all supervisors were either equipped with smartphones/tablets or their personnel phones had the open data kit (ODK) installed and configured to download the electronic forms (e-forms) for electronic data collection and transmission. In each state, the WHO state EPI officer, McKings consultant, STOP consultants, UNICEF, and other partners supported the state MOH officials in the preparation and implementation of the campaign.

**Study design:** pre-campaign activities started a month before the commencement of the campaign with each state updating and sharing the status of implementation of activities (these activities include microplanning, team selection, training, community mobilization, and sensitization, etc.). A google spreadsheet was utilized to update and monitor the status of implementation by states, which could be viewed at the national level to decide if a state or county is ready to implement. The training was conducted at all levels (national, state, county, and payam) with the lowest level of personnel being the team supervisors. A total of 285 supervisors and monitors teams were trained using mobile smartphones and tablets on ODK collect configuration, installation, and downloading of the e-checklists and were assigned to areas (counties) where they will provide support to supervise and monitor the campaign (Table 1).

**Table 1:** number of supervisors trained to supervise and monitor the campaign

States	National sup	State sup	McKings cons	UNICEF sup	Independent mon	WHO field staff *	LQAs surveyors	Total
National	2		5	7				14
Lakes	6	8			31	10	4	59
Northern Bahr El Ghazal	3	5			20	6	4	38
Unity	1	9			4	11	4	29
Upper Nile	1	7			12	10	4	34
Warrap	6	7			24	9	5	51
Western Bahr El Ghazal	2	3			8	5	2	20
Western Equatoria	4	6			18	8	4	40
Total	25	45	5	7	117	59	27	285

WHO field staff*: EPI officers, stoppers, and field supervisors; sup: supervisors; mon: monitor; cons: consultant

Intra-campaign monitoring took place during the four (4) days of the campaign, with supervisors being allocated payams, where they were expected to supervise and coordinate with the support of the county and state health teams as well as other partners in the county. Each supervisor completed a team supervision checklist electronically using the ODK checklist for every team he/she supervises, conducts both inside and outside monitoring, and fills the appropriate checklist using a simple random sampling methodology to select ten (10) households in each village supervised. The data collected are sent in real-time to a central server hosted in AFRO (Brazzaville). All checklists for the campaign were embedded with coordinate location, which gives the location of all places supervised and houses visited during the campaign. The post-campaign survey took place over two days independently by CORE group immediately after the immunization campaign and a two-stage cluster random sampling design was used to select the sample and the supervisors selected twenty households from within each cluster to apply the monitoring tool using ODK. ODK Collect was the application of choice due to its ability to cater for large data expedition in collecting data on mobile phones, more tenable to low resource settings like Africa [26-28].

**Data collection and management:** the campaign checklist forms were all translated from paper format into Microsoft Excel (XLS) forms and uploaded into ODK electronically, where they were pre-tested and debugged where corrections were needed. The electronic checklists were then uploaded onto the android-based smartphones for all activities, pre-campaign, intra-campaign supervision, and post-campaign survey. The checklists were enhanced with options of taking geo-coordinates location with in-built constraints and skip logics for easy flow and addressing errors at the point of entry, and validation with immediate identification of areas with poor indicators for ease of follow up. All supervisors and monitors (PCE and LQAs) household interviewers received appropriate training on the usage of the tool and interview techniques. The administration of data was agreed to be limited to the WHO data team for quality control, monitoring, and analysis of data, however, MOH and partners were able to view all the datasets. Data were transmitted in real-time as soon as the survey was completed from the field in places with good internet connection. The ODK collect can work in both real-time and offline, hence places where the internet and communication were a challenge, the users saved the forms and later sent them to the server when internet connectivity became available, but the greater emphasis was made for data to be transmitted in real-time to ensure that programme officers can monitor the progress of the campaign and take decisions where necessary.

The intra-campaign indicators had the components of team supervision, which looked at the performance of the team, it had several indicators that were being monitored to guide and take corrective action as the campaign was going on. The other aspect was the in-process monitoring for both inside and outside households, where children were sampled to find areas/locations and households with missed children. The lot quality assurance sampling (LQAs) was used to evaluate the campaign alongside the post-campaign evaluation (PCE), the LQAs result was used to identify locations where the campaign had failed using the decision rule on the number of children missed in that location. The indicators were eventually disaggregated down to the settlements where the children are missed with the coordinate location of the household of these children.

**Figure 1:**
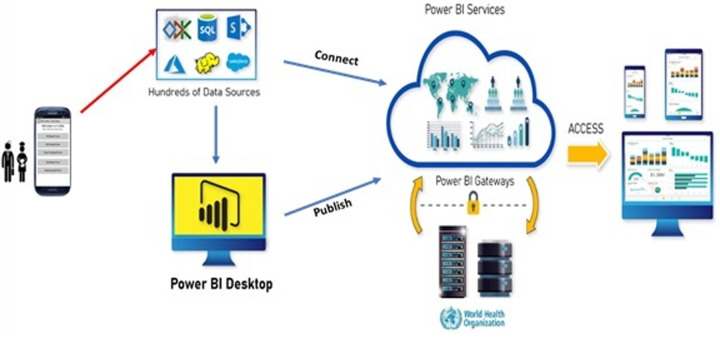
architecture of the system showing the components and connection flow

**Data analysis:** Power BI was employed to build reports and dashboard, which was connected to the ODK server to fetch the data in real-time (Figure 1), with a visualization that was customized and filtered to highlight the status of the campaign and showcase coverage and the quality of the outbreak response. The report was then published as a web link, with all partners given access to monitor the performance of the campaign in real-time. An automated schedule was set up to refresh the data from the server four (4) times a day during the campaign at different intervals (9 a.m., 12 p.m., 3: 30 p.m., and 6: 00 p.m.) to ensure that the most up-to-date information is reflected on the dashboard. Feedback from the dashboard was used during the daily meeting at the national emergency operation center (EOC) during the campaign, and details of the dashboard report were discussed, and appropriate decisions and actions are taken using the information available to the ministry of health and all partners.

The time duration from the time data was collected from the field to the time it reached the national level was calculated using the time stamp feature, which is embedded in the ODK data collection tool. This allowed us to monitor when the data collection starts, ends and when it eventually reached the server for download at all levels. The time duration for the paper-based method was obtained from previous analysis conducted manually to determine the number of days taken to get the data at the national level from the field. However, the administrative data (total children immunized by states, counties, and payams) disaggregated by different age groups were entered into an excel sheet and uploaded onto the Power BI. This is because there were no enough mobile phones at the payam level to allow for these data to be captured in an online, and the unavailability of power and internet services at some locations within the country.

**Figure 2:**
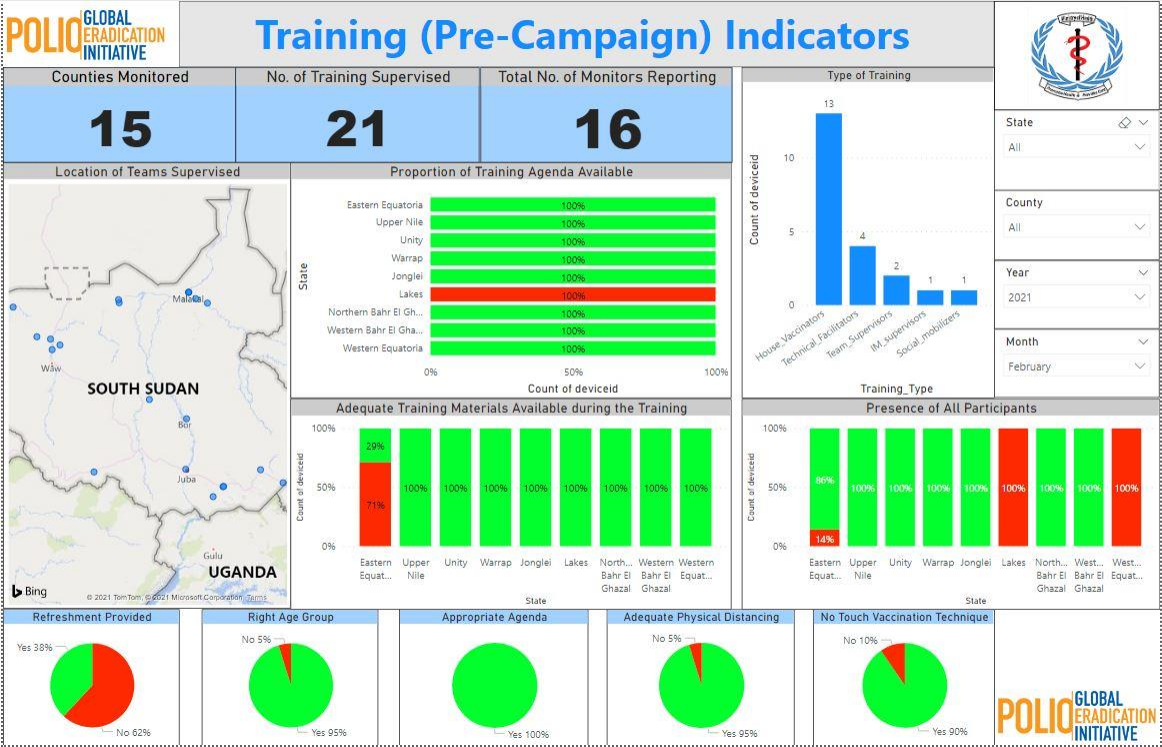
indicators showing pre-campaign indicators (training supervision checklist)

The dashboard report developed catered to both pre, intra, and post-campaign activities, with indicators selected to monitor the quality of the campaign. A total of seven (7) feedback pages formed the dashboard report covering the entire campaign indicators, each page had visualizations that monitored the selected indicators. The pre-campaign indicators used were preparedness validation and training components, which assess indicators related to the readiness of each cluster/state in starting the campaign, it also had indicators that monitored the quality of the training conducted at both levels before the start of the campaign (Figure 2). The report also depicts the proportion of children with finger marked, and children not finger marked (missed), with the reasons why children are missed based on the different categorizations that were considered as options in the checklists. All the feedbacks available on the dashboard report have been stratified by states, counties, payam, and down to the health care centres, to allow programme managers to narrow down to the lowest level of the system for corrective actions to be taken.

## Results

The duration (time taken to receive data) of data transmission from the field to the national level has improved for both the different phases of the campaign (pre, intra, and post), with a P<0.001 for all the data sets compared. Table 2 summarized the different data sets for the three (3) phases of the campaign, with an average duration for both paper-based in comparison to the use of electronic-based (ODK), a paired t-test was carried to test for significance. Table 2 depicts the comparison between the average time taken between the paper-based and electronic data timeliness of reporting. The pre-campaign data sets (training and preparedness validation) had an average of 58 hours from collection to the national level for action for paper-based, with an average of 5 hours for the electronic-based tool, showing a significant difference (P<0.001). The intra-campaign data used (team supervision and inside/outside monitoring) showed an average of 178 hours using the paper-based system, with 5 hours for the electronic-based system, giving a significant difference (P<0.001). In the same vein, the post-campaign data (LQAs) had equally shown a significant difference between paper-based and electronic-based (P<0.001) timelines for reporting. However, the number of records on the server for the training checklist was minimal as compared to expected, since this is the first time the strategy was used, as most of the personnel in the states and counties conducted the pre-implementation activities without using the mobile phone, but the few records available on the server were used for the analysis and populating the dashboard.

**Table 2:** comparison of timeliness of data reporting comparison between paper-based and electronic-based (using ODK) from field to national

Campaign phase	Data sets	Paper-Based - (avg time in hours)	Electronic-based - (avg time in hours)	Std. deviation	Std. error mean	95% confidence interval of the difference		P-value
						Lower	Upper	
Pre-campaign	Training checklist	58	5	13.322	5.035	40.536	65.178	0.001
	Preparedness validation	58	5	13.322	5.035	40.536	65.178	0.001
Intra-campaign	Team supervision	178	5	13.322	5.035	160.54	185.18	0.001
	In-process monitoring (inside/outside)	178	5	13.322	5.035	160.54	185.18	0.001
Post-campaign	LQAs	86	8	22.026	8.325	57.487	98.228	0.001

**Figure 3:**
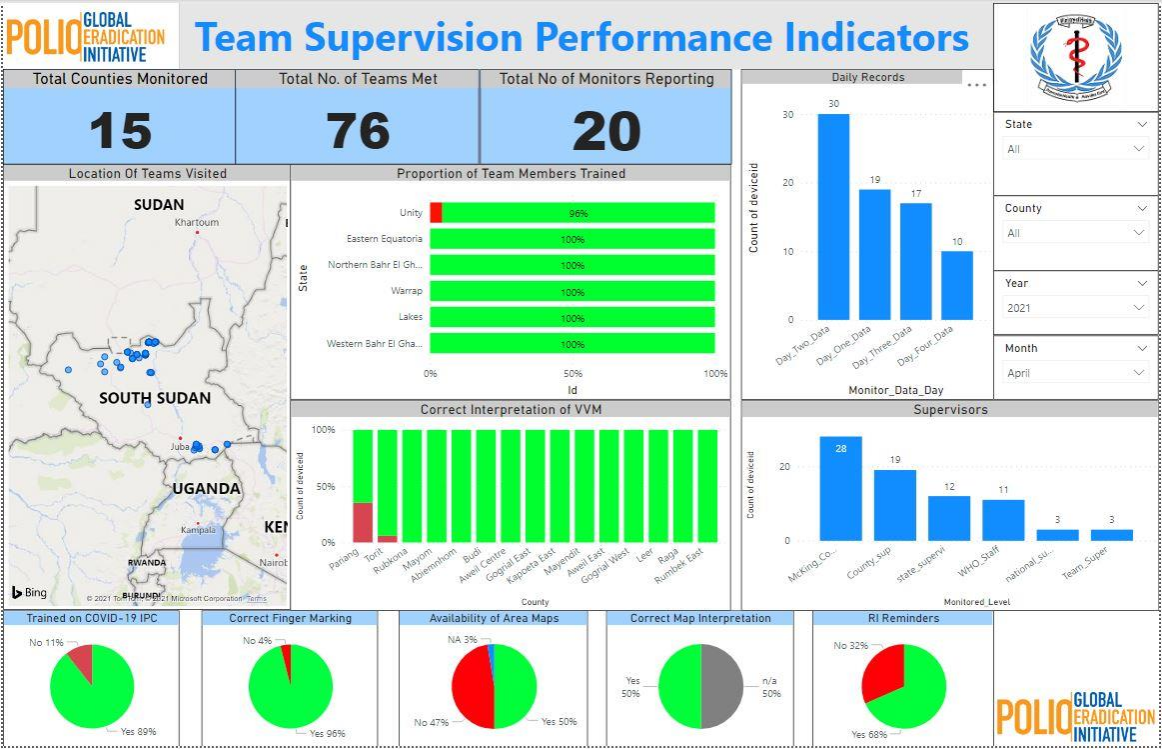
indicators showing team supervision indicators (intra-campaign)

The training indicators monitored by the dashboard include the total number of trainings that were supervised at all levels (national, state and counties), and the number of supervisors that monitored the trainings. Some of the key indicators for the training were the availability of agenda, training materials, presence of all participants invited, provision of refreshment during the training, appropriate agenda, etc. (Figure 2). Figure 3 portrays the team supervision checklist, which highlights the performance of the teams while in the field. Some of the indicators monitored by the dashboard included the proportion of team members trained, proportion of team members who know how to interpret the vaccine vial monitor (VVM) correctly, teams of area maps available and know how to interpret the maps correctly to guide their work, and teams that were marking fingers correctly in the field, etc.

**Figure 4:**
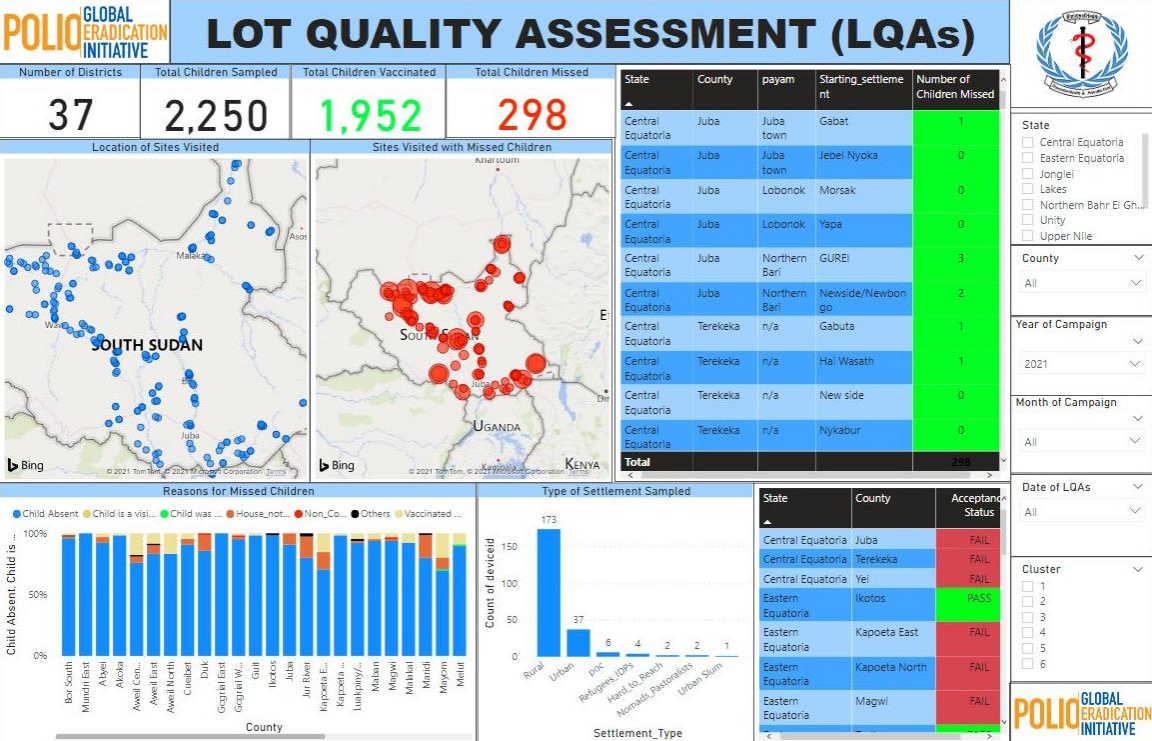
lot quality assurance sampling (LQAs) indicators showing indicators for monitoring campaign quality

Other indicators monitored included the availability of vaccines in the field and the status of the vaccine (vaccine vial monitor (VVM)), this allowed for issues to be addressed instantly when the supervisors were in the field. For instance, in previous campaigns, when payams ran out of supplies (vaccine or devices), they had to send a request to the counties and states at the end of the day before these are replenished, but using this tool, shortages of supplies at the team level were identified and escalated to the right level (payam/counties), immediately the data was uploaded where corrective action could be taken and documented by the appropriate level. Figure 4 highlights the lot quality assurance sampling result, which is used to monitor the quality of the campaign. It showed the number of children sampled by the surveyors, the total number of children vaccinated, total children missed, reasons for missing these children and the location where they are missed, etc.

## Discussion

The use of these tools has shown to improve the timeliness of data reporting, and the time taken to get the data to the national level for both pre, intra and post campaign data (Table 2), all the different data sets showed that it is statistically significant (P<0.001) for all the data sets compared between the paper-based and the electronic-based using ODK. This is in agreement with studies, which showed that the use of mobile phone electronic data collection improves timeliness, accuracy, and increases data availability [29-32]. Consequently, the post-campaign data for LQAs surveyors had improved with the final status of county acceptance level visible in real-time (Figure 4), as against previous campaigns where the data has to be downloaded, analysed before a presentation is made, and shared with all stakeholders. These had helped monitor places with missed children (including coordinates location) to allow the team to plan for revisit targeting these missed children and can also be used to plan strategies that may adequately ensure good coverage in these locations in subsequent rounds. Data availability had improved and the country is now able to meet up with the expected deadline for submitting LQAs data to the African regional office (AFRO) rapid response team (RRT) within 24-48 hours after the campaign as stipulated in the cVDPV outbreaks response guideline [4].

The use of a real-time dashboard and data transmission during the intra-campaign monitoring activities helped tremendously in identifying problems on time towards addressing them while supervisors are already in the field throughout the campaign. The tool facilitated real-time analysis of disaggregated data up to the payam, and village level, especially with the team supervision checklist where details of each team supervised and the location where they worked are visible to all stakeholders (Figure 3). The tool equally highlighted processes and challenges that the teams faced in the field and allowed for critical challenges to be escalated to the next level for a prompt solution, and for corrective action to be taken on the spot during the campaign effectively (Figure 3). One critical component that the tool addressed is the issue of transparency across all stakeholders and partners supporting the campaign due to improved access to data and information in real-time which foster communication, collaboration and follow up between different program managers and partners at the national and states level where decisions to guide the campaign are taken. The result of our study is also in line with similar findings on adapting mHealth solution in health services and quality of care [33,34].

To the best of our knowledge, this is the first time that real-time monitoring of all the phases and indicators of a polio campaign is being conducted in South Sudan [21]. Our study demonstrates that the use of this tool during this round of campaign had enhanced the transparency of data management and the reporting timeliness of the data from the field to the national level since the system had successfully truncated the components of data collation, collection, entry, and analysis at the field level through directly sharing of the data from the phone to the server (where internet is available), and these had equally increased the potential of informing programme officers and policymakers across all stakeholders on the programme planning and programming that support decision-making for immediate action in both pre, intra, and post-campaign modes, this validates similar studies that opined the use of mobile phones in improving the timeliness of data reporting [19,35,36]. It is also worth mentioning that during the conduct of this campaign, there was a daily feedback meeting, and using data from the dashboard allowed the program to take decisions and actions informed by real-time data. Previous campaigns do not have consistent meetings where feedback was provided based on data from the field, due to the limitation of application for real-time data transmission. Hence, corrective action could not be made on time for additional intervention.

Some of the limitations of the study are that this tool does not automatically fetch the post-campaign evaluation (PCE) data for inside and outside monitoring, which is currently being handled by another agency (CORE-group) because they used a server different from the one used to populate the dashboard, and the tally administrative data is captured manually using an Excel workbook. Hence, both data from PCE and the tally sheets are received in an Excel form and uploaded into the Power BI report to populate the dashboard towards ensuring having a one-stop point for all information about the campaign. However, plans are underway in moving the tally administrative data into the ODK platform where it can be directly entered from the county and payam level to allow for automatic update, and the link to the PCE data will be provided by CORE-group to be fetched directly into the dashboard. It is equally important to acknowledge that this study only focuses on the development and use of the real-time interactive tools to enhance the decision-making processes during the campaign processes, and not the results of the campaign, hence the need for additional studies that will monitor the outcome of the campaign. Consequently, we noted the lack of internet availability in some locations of the country, leading to delay in data transmission from the field in real-time, however, provisions were made to save the data on the phone and later transmitted wherever the internet becomes available.

## Conclusion

Our findings from this study had demonstrated that the use of a real-time interactive visualization dashboard had improved the conduct of polio campaign processes, with the potential of improving the outcomes towards ensuring timely information, accountability, and transparency across different partners and stakeholders. It can be applied not only to the polio immunization programme, but all other similar campaigns like Measles, Cholera, etc. which require timely information to support decision-makers in guiding the conduct of the activities. In South Sudan, the implementation of this tool had helped the global polio eradication initiative (GPEI) partners and the ministry of health to identify achievements, gaps, and challenges in reaching children in the field, and support in identifying locations with missed children promptly to appropriately plan for means of reaching these children, in ensuring all children are reached with the polio vaccines. The study has also demonstrated the value of ODK linked with Power BI in ensuring real-time data transmission for supporting campaign monitoring processes (pre-, intra, and post), and we encourage further studies that will compare the effects of this tool on the outcome of the campaign (coverage).

### What is known about this topic

- Digital health technologies had proven to improve real-time data collection and monitoring to support health programmes;
- Digital health technologies and geographic information services (GIS) have been deployed to support the conduct of immunization campaigns in the polio programme;
- The use of ODK for data collection and linked to Power BI dashboard for visualization can enhance real-time monitoring of campaign indicators during outbreak response activities.

### What this study adds

- The use of real-time monitoring of campaign indicators had improved the timeliness and completeness of data reporting during the campaign;
- The use of the tool had shown that gaps can be identified, and challenges can be resolved while in the field and had helped identify houses and settlements with missed children;
- Our study portrays the capability of using ODK in complex and humanitarian settings to collect data in the field and linked to Power BI for analysis and monitoring results.
