# Erratum: Real-time monitoring of a circulating vaccine-derived poliovirus outbreak immunization campaign using digital health technologies in South Sudan doi: 10.11604/pamj.2021.40.200.31525

**DOI:** 10.11604/pamj.2022.41.105.33607

**Published:** 2022-02-07

**Authors:** The Pan African Medical Journal

**Affiliations:** 1 The Pan African Medical Journal, Nairobi, Kenya

**Keywords:** Erratum, Geographic information system (GIS), circulating vaccine derived poliovirus (cVDPV), vaccination, open data kits (ODK), Power BI

## Abstract

This Erratum modifies article by Isah Mohammed Bello, Maleghemi Sylvester, Melesachew Ferede, Godwin Ubong Akpan, Ademe Tegegne Ayesheshem, Michael Nzioki Mwanza, Samuel Okiror, Atem Anyuon, Olu Olushayo Oluseun: “Real-time monitoring of a circulating vaccine-derived poliovirus outbreak immunization campaign using digital health technologies in South Sudan” and its publication reference i.e. Pan African Medical Journal. 2021;40:200. Access corrected manuscript Pan African Medical Journal. 2020; 40: 200. Access corrected manuscript *Pan African Medical Journal. 2021; 40: 200*. doi: 10.11604/pamj.2021.40.200.31525.

## Erratum

The original version of this article [1] had authors' names reversed in the article citation on PubMed. The authors' names Maleghemi Sylvester and Olu Olushayo Oluseun have been corrected by inverting authors' names as follow: “Sylvester Maleghemi” and “Olushayo Oluseun Olu”.
